# Breast carcinoma originating from a silicone granuloma: a case report

**DOI:** 10.1186/s12957-015-0509-6

**Published:** 2015-02-22

**Authors:** Ryoichi Nakahori, Ryuji Takahashi, Momoko Akashi, Kana Tsutsui, Shino Harada, Roka Namoto Matsubayashi, Shino Nakagawa, Seiya Momosaki, Yoshito Akagi

**Affiliations:** Department of Breast Care Center, National Hospital Organization Kyushu Medical Center, 1-8-1 Jigyohama, Chuou-ku, Fukuoka Japan; Clinical Research Institute, National Hospital Organization Kyushu Medical Center, 1-8-1 Chigyouhama, Chuou-ku, Fukuoka Japan; Department of Pathology, National Hospital Organization Kyushu Medical Center, 1-8-1 Chigyouhama, Chuou-ku, Fukuoka Japan; Department of Surgery, Kurume University School of Medicine, 67 Asahi-machi, Kurume, 830-0011 Kurume, Japan

**Keywords:** Liquid silicone injection, Breast augmentation, Breast cancer

## Abstract

Breast carcinoma rarely occurs in cases of foreign body granulomas following liquid silicone injection. Although the Food and Drug Administration (FDA) banned the use of all silicone injection products in 1992, liquid silicone injection for breast augmentation continues to be performed illegally. We herein report a case of breast carcinoma following liquid silicone injection in a 67-year-old female.

A total of 45 years after liquid silicone injection, the patient had felt a breast mass in the right breast. Mammography showed a smooth mass that retracted the right nipple. Due to the presence of a marked acoustic shadow caused by the granulomas, evaluating the mass on ultrasonography was difficult. However, magnetic resonance imaging (MRI) showed a lobulated mass under the right nipple. The mass exhibited low signal intensity (SI) on T1-weighted images and intermingled high and low SI on T2-weighted images. Heterogeneous early enhancement with central low intensity was noted on dynamic contrast-enhanced MRI. Several oval-shaped low SI structures in the adipose tissue and disruption of the pectoralis major muscle were also observed. We diagnosed the patient with invasive ductal carcinoma based on a stereotactic-guided Mammotome® (a vacuum-assisted biopsy system manufactured by DEVICOR MEDICAL JAPAN, Tokyo, Japan) biopsy and subsequently performed mastectomy and axillary lymph node dissection (with a positive result for the sentinel node biopsy). Histologically, invasive ductal carcinoma was observed in the silicone granuloma.

The development of foreign body granulomas following breast augmentation usually makes it difficult to detect breast cancer; thus, various devices are required to confirm the histological diagnosis of breast lesions. The stereotactic-guided Mammotome® biopsy system may be an effective device for diagnosing breast cancer developing in the augmented breast.

## Background

Liquid silicone injection for breast augmentation was initiated worldwide and in Japan in the 1940s [1,2]. However, due to complications such as inflammatory changes and fibrosis, the use of liquid silicone for breast augmentation has been decreasing [3,4]. In August 1991, the Food and Drug Administration (FDA) prohibited the marketing or sale of injectable liquid silicone for esthetic purposes [5]. Notably, the FDA has never approved the use of injections of liquid silicone for cosmetic treatment in patients. In 1992, the FDA officially banned the use of all silicone injection products in medical procedures. However, liquid silicone injection for breast augmentation continues to be performed illegally, making it difficult to estimate the number of females who have received this procedure [3,4,6]. We herein report a case of breast carcinoma following liquid silicone injection in a 67-year-old female.

## Case presentation

**Figure 1:**
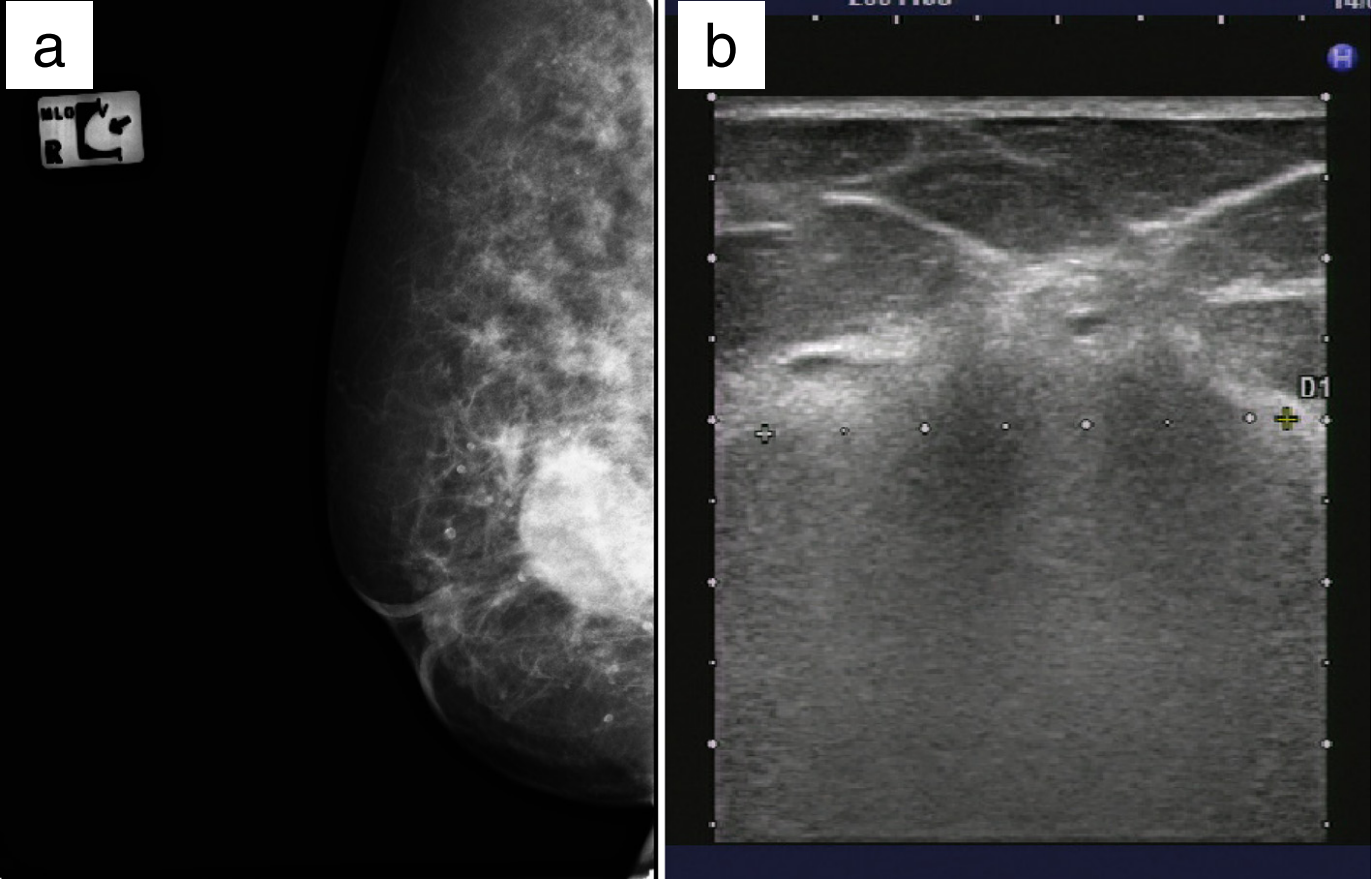
**Mammography and ultrasonography. (a)** Mammography showed a smooth mass that retracted the right nipple. **(b)** Ultrasonography revealed a so-called ‘snowstorm’ appearance with diffuse hyperechogenic lesions and posterior shadowing.

**Figure 2:**
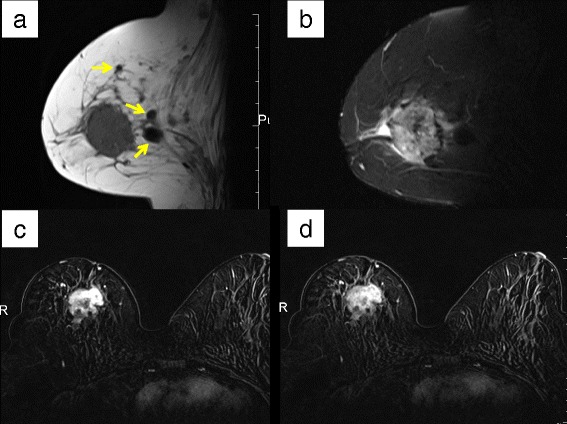
**Breast magnetic resonance imaging (MRI). (a)** T1-weighted MRI showed a low signal intensity (SI) mass (3.5 × 3.2 × 4.0 cm in size) that retracted the right nipple. Several oval-shaped low SI structures in adipose tissue and disruption of the pectoralis major muscle were also observed (arrows). **(b)** The mass showed intermingled high and low SI on T2-weighted images. **(c, d)** Heterogeneous early enhancement with central low intensity was noted on dynamic contrast-enhanced MRI (c: early phase, d: delay phase).

A 67-year-old female felt a breast mass in her right breast and visited our hospital. She had received silicone oil injection into bilateral breasts at 22 years of age. Mammography showed a smooth mass that retracted the right nipple (Figure 1a). Ultrasonography revealed a so-called ‘snowstorm’ appearance with diffuse hyperechogenic lesions and posterior shadowing (Figure 1b). It was difficult to distinguish between the silicone granuloma and breast cancer using these modalities; therefore, magnetic resonance imaging (MRI) was performed. T1-weighted images showed a low signal intensity (SI) mass (3.5 × 3.2 × 4.0 cm in size) that retracted the right nipple (Figure 2a). Several oval-shaped low SI structures in the adipose tissue and disruption of the pectoralis major muscle were also observed (Figure 2a). The mass showed intermingled high and low SI on T2-weighted images (Figure 2b), while heterogeneous early enhancement with central low intensity was noted on dynamic contrast-enhanced MRI (Figure 2c,d). On diffusion-weighted images, the mass exhibited high SI due to the restricted diffusion.

**Figure 3:**
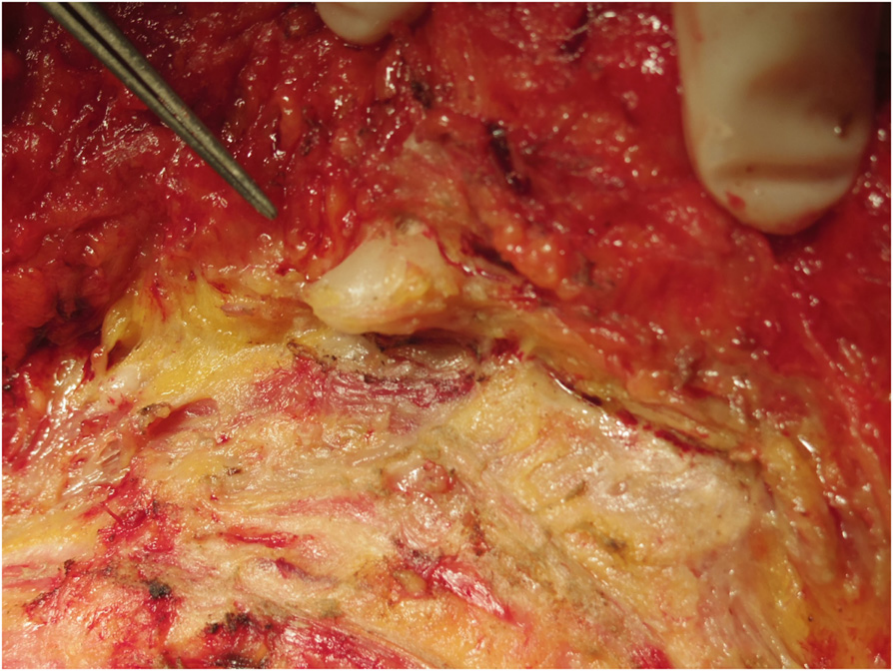
**Intraoperative findings.** We performed mastectomy and axillary lymph node dissection (a positive result for the sentinel node biopsy). The intraoperative findings showed marked adipose degeneration in the retromammary space and many white round bodies indicating silicone granulomas.

**Figure 4:**
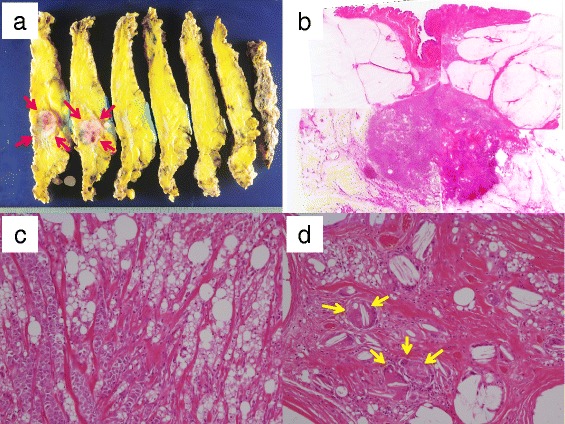
**Resected specimens and histological findings. (a)** The resected specimens showed a white and partially red lobulated mass (arrows). **(b, c)** Histologically, stromal invasion of invasive ductal carcinoma was observed in the silicon granuloma (hematoxylin & eosin [HE] stain; b: HE stain X4, c: HE stain X100). **(d)** Inflammatory granulomas and foreign body giant cells (arrows) were observed (HE stain X100).

We investigated available approaches for diagnosing this tumor histologically and consequently performed a stereotactic-guided Mammotome® (a vacuum-assisted biopsy system manufactured by DEVICOR MEDICAL JAPAN, Tokyo, Japan) biopsy because the tumor was clearly detectable on mammography. The most high-density area of the tumor was targeted in order to obtain tissue samples from the tumor. According to the histological findings of the biopsied specimens, we diagnosed the patient with invasive ductal carcinoma and subsequently performed mastectomy and axillary lymph node dissection (with a positive result for the sentinel node biopsy). The intraoperative findings showed marked adipose degeneration in the retromammary space and many white round bodies, which indicated the presence of capsulated silicone (Figure 3). The resected specimens demonstrated a white and partially red lobulated mass (3.4 × 2.7 cm in size) (Figure 4a).

Histologically, stromal invasion of invasive ductal carcinoma was observed in the silicone granuloma (Figure 4b,c), and inflammatory granulomas and foreign body giant cells were also observed (Figure 4d). The pathological diagnosis was as follows: scirrhous carcinoma, nuclear grade 3, positive for lymph node metastasis (1/18), estrogen receptor-positive (95%), progesterone receptor-positive (30%), human epidermal growth factor receptor 2 (HER2)-negative (score 1+ on immunohistochemistry), and a high Ki-67 index (35%). Four cycles of treatment with EC (90 mg/m^2^ of epirubicin and 600 mg/m^2^ of cyclophosphamide) were given as adjuvant chemotherapy, and the patient currently remains alive, without recurrence, at 10 months after the surgery.

## Discussion

As a result of liquid silicone injection to the breast, many females develop inflammatory changes and granulomas, which complicate breast cancer screening [7]. Mammography in these patients often shows numerous bilateral round or oval masses with rim calcification, while ultrasonography shows a ‘snowstorm’ appearance with diffuse hyperechogenic lesions and posterior shadowing characteristic of free silicone, ring-down artifacts [8,9]. These radiographic findings make it difficult to detect breast cancer in the early stage; thus, contrast-enhanced MRI is beneficial for breast cancer screening [10,11]. Many cases of breast carcinoma developing in the augmented breast have been reported to date [6,10-23]. Major cohort studies investigating the frequency of breast cancer following breast augmentation have reported rates ranging from 0.2% to 2.7% [24]. We herein reported a case of breast carcinoma that developed in the augmented breast following liquid silicon injection. Histologically, inflammatory granulomas and foreign body giant cells were observed with stromal invasion of invasive ductal carcinoma. Various histological types of cancer have been reported after liquid silicone injection in the previous literature [6,12,15,17,18,20-23]. Interestingly, there are two cases of squamous cell carcinoma following liquid silicone injection [15,17]. Handel *et al.* mentioned that augmented patients present with a statistically greater frequency of palpable lesions, with a slightly greater risk of invasive tumors and an increased likelihood of axillary lymph node metastases [13]. Despite this observation, there are no statistically significant differences in the stage at diagnosis or the prognosis between augmented and non-augmented patients [13,19]. However, most of the augmented patients in these studies received a bag prosthesis or paraffin injection. Therefore, the details of the histological and clinical characteristics of breast cancer following liquid silicone injection have not been thoroughly investigated to date.

Performing the accurate detection and evaluation of breast carcinoma in cases of foreign body granulomas is difficult, as the images are severely affected by artifacts caused by the granulomas. Therefore, various devices are required to confirm the histological diagnosis of breast cancer. In the present study, we performed a stereotactic-guided Mammotome® biopsy because the tumor was detectable on a mammogram in this case. Importantly, the early diagnosis of breast cancer resulted in successful curative surgery and subsequent adjuvant chemotherapy. The stereotactic-guided Mammotome® biopsy system may be an effective device for diagnosing breast cancer developing in the augmented breast.

## Conclusions

In conclusion, obtaining an early diagnosis of breast carcinoma originating from silicone granulomas is difficult. The stereotactic-guided Mammotome® biopsy system may be an effective device for diagnosing breast cancer developing in the augmented breast.

## Consent

Written informed consent was obtained from the patient for publication of this case report and any accompanying images. A copy of the written consent form is available for review from the Editor-in-Chief of this journal.

## Abbreviations

FDA: Food and Drug Administration
MRI: magnetic resonance imaging
SI: signal intensity
DWI: diffusion-weighted images
HER2: human epidermal growth factor receptor 2
HE stain: hematoxylin & eosin stain
